# What can the COVID-19 pandemic tell us about the connection between media and religion? The case of the Seventh-Day Adventist Church in Poland

**DOI:** 10.1007/s41682-021-00091-z

**Published:** 2021-10-28

**Authors:** Marta Kołodziejska

**Affiliations:** The Institute of Philosophy and Sociology, Polish Academy of Sciences, Warsaw, Poland

**Keywords:** Deep mediatization, The Seventh-Day Adventist Church, COVID-19, Digital media, Religion

## Abstract

The aims of this paper are to investigate 1) how the Seventh-Day Adventist Church in Poland has reacted to the COVID-19 pandemic restrictions and lockdowns imposed nationwide, 2) how the pandemic context has influenced the Church’s digital media productions, and 3) how the Church has adapted to some of the trends and consequences of deep mediatization (Hepp et al. 2017, 2018).

Based in the constructivist concept of deep mediatization (Hepp et al. 2017, 2018), the paper analyzes interviews with Seventh-Day Adventist media professionals from Poland within the framework of the Sociology of Knowledge Approach to Discourse. Additionally, selected official YouTube channels of local congregations in Poland are analyzed quantitatively to identify the changes in the number of uploads and views.

The analyses show that as a result of the COVID-19 lockdowns and restrictions, media production and use increased substantially in some digital media formats produced by the Seventh-Day Adventist Church, which was on the one hand a response to the pandemic context as such, and on the other hand, a reaction to the discriminatory laws which drastically limited the Church’s activities offline. The pandemic has opened up new possibilities of participation, but also increased the chances of digital divides and exclusions.

The study concludes that to mitigate the risks related to digital divides during the pandemic, some Polish pastors took on the roles of media experts and educators, incorporating technical skills in their authority status. This suggests that transformations of religious authority may be another consequence of deep mediatization processes.

During the COVID-19 pandemic, most European states imposed various lockdown measures, temporarily limiting businesses, and restricting public gatherings. For Churches and religious communities, this meant limiting the number of attendees during religious service, cancelling events, offline meetings of committees, councils, and informal groups. They also had to face new problems, or experiences them on a larger scale: with hundreds of people suffering physically and mentally from the illness, isolation, and other COVID-related issues, facing unemployment or death of their loved ones, the Churches often had to intensify their support-oriented activities. This included anything from doing the groceries for elderly citizens, distributing free meals, to providing online counseling, to name just a few examples. Since regular meetings were put on hold or severely limited, most Churches had to resort to digital media to continue their activities and maintain the community.

In Poland, all religious institutions were faced with the new reality, and the uncertainty related to the development of the pandemic. However, the dominant Roman Catholic Church (RCC) was in a different position than the minority Churches: it has considerably more resources, a developed and varied media infrastructure, and it is favoured by the ruling party, Law and Justice (PiS), who funds various Catholic organizations. Small minority Churches, like the Seventh-Day Adventist Church, have found themselves in a different position. With about six thousand members in Poland, this Protestant minority Church could not rely on public monies for financial support and had to make use of the available resources. It also had to face consequences of lockdowns and restrictions that the majority Church did not.

This paper, based in the theoretical framework of deep mediatization (Hepp et al. 2017, 2018), will investigate the implications of nationwide restrictions in Poland for the Seventh-Day Adventist Church, and will argue that due to their discriminatory effect, the restrictions have caused the Church to rely more on its digital media to maintain community, but at the same time revealed the challenges of user segmentation and digital divides among the members. It will also argue that this increased reliance on digital media is an indicator of a greater development: the intensification of the processes of deep mediatization (Hall and Kołodziejska 2021), which encompasses religion as well.

## Deep mediatization

Among various approaches to mediatization, two seem to have gained most attention in social sciences: the institutional, proposed by Stig Hjarvard (2011), and the constructivist, developed mostly by Friedrich Krotz, Andreas Hepp et al. Both concepts acknowledge the importance of media in society and the increasing connection between media and various areas of the social world, such as politics, family life, or religion. However, while the institutional perspective views the media as a separate institution with its own logic, the constructivist one considers all spheres of the social world to be increasingly entangled with the media. From the institutional viewpoint, the key process of mediatization is that various institutions, communities, and in general segments of the social world become determined by and dependent on the logic of the media. For Krotz, Hepp, and other proponents of the constructivist perspective, the key process is about the media becoming inherent part of every social domain, not only on the levels of institutions, organizations, etc., but on the most basic level of communication. Therefore, when talking about mediatization, Krotz and Hepp understand it as a metaprocess “of a communicative construction of socio-cultural reality” (Hepp 2013, p. 54), that shapes the social world on all levels (cf. Krotz 2007, 2009). This transformation should be investigated on the level of media use, i.e. what individuals, groups, and institutions do with the media, how, and for what purposes.

Deep mediatization points to the fact that due to rapid development of digital media, there has been an intensification of the processes of mediatization, which results in the digitalisation and connectivity of “old” media, and the emergence of “new ones”, and means that the formation of any part of society is related to the “technologically based media of communication” (Hepp et al. 2017, p. 14). Deep mediatization is of “cross-media, multifaceted and reflexive character” (Hepp et al. 2017, p. 13), and it is a non-linear process. One can discern several trends of deep mediatization that affect organizations, groups, and individuals (Hepp et al. 2017, p. 17):

1. Differentiation of technologically based media of communication—which points to the increase in and variance of available media, their functionalities, and media-related technologies.
2. Increased connectivity of and through media—indicating that global, cross-media communications are becoming easier and more accessible.
3. Rising omnipresence of media—which points to the fact that various human activities, which have not been previously related to media to such an extent (like face to face meetings), are becoming increasingly mediatized (holding meetings via online communicators is nothing unusual nowadays, neither is browsing social media sites when spending time with people, etc.). One can connect with others anytime, anywhere, transcending spatial and temporal boundaries.
4. The emergence of new media and rapid pace of innovation, which refers to technological development, but also new trends of media use.
5. Datafication—indicating that life becomes increasingly connected with and represented through computerized data.

These trends should not be viewed as occurring separately or in a sequence; instead, all five of them, considered together, are reflective of the changes occurring due to deep mediatization. Each of these trends may have numerous consequences for different social domains. Differentiation may result in optionality, social contingency, and new chances of participation, and increased connectivity may result in spatial extension of social domains, but also further blurring of boundaries between domains. The consequences of rising media omnipresence are the acceleration and immediacy of social processes, while the rapid pace of innovation may result in the perceived pressures to adjust to that pace on the one hand, and the exclusions, segmentations, and divides on the other hand. Datafication may result in the disguise of agency through technical intermediaries, as well as in the stabilizing of sociality, and the emergence of new possibilities of control (Hepp et al. 2017). It must be added that the trends themselves do not determine the consequences: for instance, rapid pace of innovation may open up new chances for participation, depending on the area of the social world it concerns, and the level of analysis. Due to limited space, the paper will focus on the new chances for participation, and segmentations, exclusions, and divides associated with the pandemic-related intensification of deep mediatization processes in the Seventh Day Adventist Church in Poland.

New chances for participation point to the fact that with the development of digital media, individuals and groups may gain new “access points” to various fields of the social world. For instance, tools enabling remote work may help new parents retain their job or find new employment; using online communicators by migrants allows them to keep in touch with friends and family abroad; using dating apps may be a good way to find romantic relationships at any age, and so forth. On the other hand, the trends of deep mediatization may lead to segmentations and divides. Digital divides (cf. Norris 2001; Tsatsou 2011) describe unequal distribution of power related to the adjustment to digital media innovations: those who have the skills and the means to make these adjustments have advantage over those who do not. The divides and segmentations can be observed on micro, meso, and macro levels, i.e. within families, organizations, and between nation-states. While some digital technologies may be used to mitigate the divides (for instance, accessibility tools for users with disabilities), the development of others may sustain the exclusions.

Since religious organizations participate in the deep mediatization-related transformations, they inevitably have to face their positive and negative consequences. As will be shown on the example of the Seventh-Day Adventist Church (SDA) in Poland, the context of the pandemic has also brought them to light.

## Material and methods

The basis for analysis presented in this paper is the interview material gathered in late Spring 2020, as part of a research project focused on two Christian minority Churches in Poland and the UK, titled [anonymised]. In the project, digital and print media of both Churches (from 2016 to 2017) were analysed, along with interviews with the Church media professionals. The interviewees comprised individuals working in various Church media (digital, press, radio, etc.) in different capacities, from technical staff, journalists, editors, producers, writers, to media department directors, and to independent online evangelists. In this paper, only the Adventist interviews are analysed.

The rationale behind choosing this particular group was the fact that while Polish and British Church media are parts of the same organizational structure, namely the Trans-European Division, they operate independently and have their specificity, which is affected by the national, political, and cultural context. The COVID-19-related restrictions and laws have also constituted and transformed that context since 2020. The specific role and function of the Adventist media is further influenced by the national media environment, i.e. the entirety of available media at any given time (Hepp et al. 2017). For instance, in Poland the Seventh-Day Adventist Church has a regular 10-minute show on the national Polish radio, which is not the case in the UK. At the same time, the British Adventist community may easily connect with American Adventist television channels through satellite TV, or a variety of digital streaming platforms, some of which are not widely available in Poland. This difference, along with the particular social and media contexts, is assumed to be reflected in the interviews, which enables an in-depth reflection on how it may affect media productions and their content. Furthermore, following the principles of the constructivist perspective on mediatization outlined earlier, in order to investigate the mediatization-related trends and consequences, media use on the level of the institution was the vital object of analysis, hence the narratives produced by people responsible for developing the Church media on various stages of production had to be included.

There were 16 interviews in total, divided between two rounds, in February-May 2019 and April-May 2020—this paper includes only the latter. The scenarios followed the principles of episodic interviews (Flick 1997), and their analytical aim was to reveal two dimensions of knowledge among the interviewees: the semantic, which encompasses generalized and abstract assumptions, norms, and values (including those related to the Polish context, the role of the media, and Adventism as a minority Church in Poland), and the episodic, consisting in everyday, concrete experiences (such as particular events or media activities during the pandemic). The questions pertained to how different media is produced in the Church, to the structure of authority in media productions, the aims of the Church media and the productions, as well as the particular social, political, and media context in which the Church operates in Poland. In the second round, the interviewees were also asked to comment on how the COVID-19 pandemic has influenced the Church and its media, and how the institution sees its role in the global health crisis.

The interview material was then analysed with the use of the Sociology of Knowledge Approach to Discourse (Keller, 2013), which is a particularly useful framework for investigating the construction of knowledge in a three-step procedure, i.e. through the analysis of the problem structure, the connecting meaning patterns, and overarching narrative patterns (which include identification of key actors, ascribed values, storylines, and connections between them). The benefit of this approach, deployed in combination with the episodic interviews, was a comprehensive view on how both the Church media and media producers construct the knowledge and meaning of their environment, and how the different aspects of knowledge overlap, diverge, or are in dialogue with one another. SKAD acknowledges the power relations that affect the narrative patterns’ construction, but without making normative claims about the relations of dominance; rather, power structures are seen as inherent in any narratives, influencing the positions of actors. This type of focus allowed us to investigate how the role of the Church authorities may change during the pandemic.

To complement the qualitative step, key digital media channels of the Polish SDA Church^1^ were analysed quantitatively: the purpose of the segment was to determine the changes in the quantity of content published in the first year after the onset of the pandemic in Poland (i.e. March 2020–March 2021), as compared to the analogous period in 2018–2019 (see Table 1 below). Its second purpose was to note any major changes in dominant topics or style of publishing, for instance from less to more interactive, more focused on certain issues, etc.

**Table 1:** The number of video uploads and average views per video for March 2018–2019, and March 2020–2021 on selected Polish Seventh-Day Adventist YouTube channels. (Source: own elaboration)

	March 2018–March 2019		March 2020–March 2021	
Selected SDA YouTube channels	Number of video uploads	Average number of views per video	Number of video uploads	Average number of views per video
Warszawa Centrum	93	887	115	2675
Adwentyści Skoczów	5	369	166	473
Adwentyści Łódź	52	966	186	1123
Adwentyści Gdynia	3	306	27	109

As a result of the analysis, four conclusions were drawn:

1. Overall, the Church’s digital content productions have increased in some of the analysed media when compared to 2018–2019.
2. The interviewees reported greater interest in their online content in general, but the switch to digital media triggered several problems connected to access to technology among Church members.
3. The nationwide restrictions on Churches, imposed by the Polish government, although on paper equal for all religious organizations, in fact discriminated against religious minorities.
4. In reaction to this discrimination, the Seventh-Day Adventist Church undertook various actions, including addressing the government in open letters, and keeping the intensive pace of digital media productions throughout the analysed period.

Those conclusions indicate that as a result of the COVID-19 lockdowns and restrictions, digital media production and use increased substantially within the Church, which on the one hand was a response to the pandemic context as such, and on the other hand, a reaction to the discriminatory laws which drastically limited the Church’s activities offline.

## Poland in 2020: Churches during lockdowns

In March 2020, Poland had its “patient zero” infected with the new coronavirus. In a few weeks’ time, the whole country was under lockdown. As part of the new regulations, the limit of the number of participants of religious services was five persons per service. A month later, that limit was changed to one person per 15 square metres in a building, and no more than 50 persons during funerals. In the summer, when the daily numbers of new COVID-19 cases were low, the restrictions were lifted nationwide, only to return in autumn with the second wave of the pandemic. The restrictions imposed in October 2020 were changing dynamically, but overall were based on the distinctions between three zones, green, yellow, and red (which depended on the number of active cases) within the country. In green and yellow zones, there were no restrictions concerning the number of participants of religious services, although face masks were mandatory. In the red zones, where the number of active virus cases were the highest, that limit was 50% of the capacity of the building. For instance, if a church could hold 200 participants during one service, only 100 of them could attend. Soon, the whole country became a red zone and the strict limits were in effect in all regions. The third wave which began in early 2021 brought about new restrictions: this time, again the one person per 15 square metres limit was maintained, but this time with added mandatory distance of 1.5 m between participants, who were obliged to wear face masks (all these restrictions were posted on the official website of the Polish government, https://www.gov.pl/web/koronawirus). Apart from those restrictions, others pertained to Church festivities and events, which for the most part had to be cancelled between March 2020 and March 2021, with some exceptions during the summer months of 2020.

The regulations outlined above were binding for all religious institutions, however, while on paper they seemed to apply equally to all Churches, in fact they affected them to a varying degree. What must be added is that during lockdowns, local community and cultural centres, theatres, and sports facilities were closed or their activity was drastically limited. While for those Churches that have their own buildings this was not necessarily a problem, for small minorities that often rent out premises to hold their weekly services and/or meetings, this was a serious issue, as it left several congregations with no place to worship. As two Seventh-Day Adventist interviewees have put it:

The government, when these regulations were created, probably did not care about the needs of religious minorities. Even those fifteen meters per person is calculated for those Churches that have, so to speak, hangars. And not chapels, rented premises, where small congregations gather. (…) So the interests of the Catholic Church were taken into account, the interests of minority Churches were not. (Polish media producer, male)

In many [Catholic] churches there are several services a day, and we usually have one service, so it’s also kind of a problem now. How, what conditions should be met by the participants, apart from the sanitary regulations, meaning who should come and who shouldn’t come, should we choose alphabetically? By age, by merit? I counted yesterday, taking into account all the rooms we have, because supposedly you can include lobbies, toilets, and catechetical rooms [into the overall area of the church-MK], so we have the biggest building in W., but still only thirty-five people can come to the service, and on the list of believers there are 387 people (…). (Polish pastor, media contributor, male)

Both interviewees reflected on the restrictions, pointing out that the limit of persons per square metres inevitably favours Churches which have large buildings. The dominant Roman Catholic Church, and in some cases also the Orthodox Church (especially in the north-eastern part of the country), typically owns its temples in Poland, which are able to accommodate hundreds of people at once. For small minorities, like the Adventists, Methodists, Baptists, but also Muslims and Jews, the person-per-square-meter limit meant that only a handful of believers could participate in a service. As the second interviewee mentioned, this inevitably raised questions of selection criteria, which cannot, in a fair manner, determine who is and who is not allowed to take part. Since the SDA Church typically has one service on Saturday, it is impossible to divide the church members into groups so that everyone can attend in different time slots. Therefore, apart from a series of organizational and technical problems, the restrictions directly influenced the decisions of religious conscience regarding whether to attend a religious service (and fulfill one’s religious obligation, meet with the congregation) or not.

Failure to comply with the restrictions could result in fines whose cost (up to 7 thousand Euros) would be a serious financial strain on minorities that rely on their members for support. This brings us to another source of inequality: the Catholic church, on top of having millions of members willing to donate to their parishes, can also count on government support. This is not the case for most religious minorities, like the SDA Church:

The [Adventist-MK] Church is supported by the donations of the faithful, we don’t have any sponsoring, we are not supported by the Polish state, nor by any national or foreign organisations, so also at this moment the donations were limited (…) (Polish pastor, media contributor, male)

The interviewee then explained that typically during service, donations are collected, and the Church members are encouraged to pay via bank transfers as well. They can also support the institution by purchasing books and magazines published by the Church after the service—so when in-person attendance was limited or prohibited, the opportunities for obtaining donations have dwindled. Furthermore, bills (electricity, water, rent, etc.) still had to be paid, which became increasingly more difficult in the absence of the regular donations. If the minority organization cannot count on financial support from other sources, the restrictions negatively impact how the Church and its organizational segments (such as charities, publishing houses, radio stations, etc.) function.

## Adventist responses to restrictions

In response to this discrimination, the Seventh-Day Adventist Church in Poland undertook several actions. Firstly, in March 2020, the editor-in-chief of the Adventist magazine “Znaki Czasu”, pastor Andrzej Siciński, sent a letter to the Polish Commissioner for Human Rights (CHR), pointing out the unequal treatment of Churches through the imposed restrictions. Then, in the official email to the Prime Minister, in April 2020 the SDA Church submitted several comments to the new regulations, along with suggestions of changes, which would be better suited to the needs of minority Churches (Siciński 2020). Simultaneously, the SDA Church was in talks with the Evangelical Alliance (association of several Protestant denominations in Poland, to which the SDA Church does not belong), who also sent a letter to the Prime minister in April 2020, requesting modification of the restrictions to accommodate minority Churches (Kamiński 2020). The CHR also supported the requests made by both Churches, in a separate letter sent to the Prime minister, which addressed several issues related to the lockdown measures, including the discrimination of religious minorities. In May 2020, in a response signed by the Undersecretary of State, Mr. Błażej Poboży, the Prime Minister responded positively to the complaints of the Alliance, and confirmed that the limits would be changed (*Otrzymaliśmy Odpowiedź Na List Aliansu Ewangelicznego Do Premiera* 2020). This, however, coincided with the nationwide loosening of the restrictions due to low number of COVID-19 infections. However, during the second wave of the pandemic in autumn 2020, when the nationwide limits on religious service participation were again calculated on the basis of the person-per-square-meters ratio, the requests made by the minority religious organizations were not taken into account.

Apart from communicating with the government, as a response to the restrictions, the SDA Church began to intensively make use of digital media to communicate with its community. Some forms of this communication were novel: for instance, several meetings that normally took place in person, such as council meetings or editorial boards, had to be done remotely, via zoom, skype, etc. Another example is the annual Hosanna festival, which is a spiritual and musical event—in April 2020, it was organized in a hybrid form, with some concerts and sermons transmitted online^2^. In other cases, while the forms of communication remained the same, they intensified, as shown in Table 1. This could be observed for instance on the YouTube channels of several local congregations, which published noticeably more videos after March 2020 than before. The congregation in Gdynia published about 45 videos between 2013 and 2019, but 27 videos only between March 2020 and March 2021^3^, and in Skoczów there were approx. 110 videos between 2012 and 2020, and over 190 between March 2020 and March 2021 alone^4^. While this increase cannot be attributed solely to the pandemic, the latter (or rather, the restrictions on gatherings that the pandemic entailed) can be regarded as encouraging more frequent uploads. Indeed, one interviewee stressed that the Church staff was on a “production alert”, committed to working overtime despite difficult conditions:

I think the timing was … just right, as they say, because it was twelve hours after the announcement of the lockdown, and we’ve already started production. (…) The first line of defense are the doctors, right? Epidemiologists, and some journalists. But here, the second line was building spiritual immunity. (Polish radio and TV producer, male)

The interviewee suggested that the role of the Church during the pandemic was not to oppose or compete with doctors and epidemiologists (as some Catholic bishops did, for instance by arguing that one cannot contract COVID-19 in church^5^), but to offer support to the people, specifically by being present in the media. The interviewee later on added that several videos posted to the YouTube channel were created in such a way that they would potentially appeal to non-Adventists as well—so that whenever someone would search for specific terms on the platform, they would have a better chance of finding the Church productions. This signals that the Church media department acknowledged the importance of positioning itself within the context of a variety of competing media productions, and did so by offering responses to the audiences’ needs it identified as the most important at that particular time.

## Between new chances of participation and digital divides

The interviewees also reported that they have observed increase traffic on their sites and YouTube channels during the pandemic, as well as some new digital media use practices among the Church members:

People have this opportunity, as the online form of running the Saturday Bible school has become widespread, to get together on Zoom, right? They meet so that they can study the Scriptures with others, in their homes, in this collective way, they sit in front of TV sets to which computers are connected, they listen to the sermon. Usually, you’d listen to the sermon in the church you went to, now it’s so that when we sit at home I’ll listen first to the sermon from Warsaw Centrum, then I’ll listen to the sermon from Podkowa Leśna, and at the end I’ll listen to the sermon from Łódź Widzew, because there are three churches that organise internet transmissions. (Polish pastor and media contributor, male)

There are no official services anywhere, so all those who can’t attend, but who want to take part, take part in one of these three broadcasts, and it happens so, we know that some people watch everything, one after another, they now they listen to services in Warsaw and in Łódź, and it works quite nicely. (Polish pastor, media contributor, male)

It seems that while listening to multiple online sermons on Saturday has been practiced by some Adventists before 2020, the pandemic context encouraged more of them to do so on a regular basis. While concrete numbers were not given, this observed change of media habits of some practitioners may be a sign that when offline community could not gather, its online extensions compensated for this lack with accessibility. In other words, the digital tools were used to maintain the sense of togetherness and belonging, in spite of the physical isolation.

As the quotes above suggest, the switch to digital media opened up new chances of participation, which is one of the consequences of deep mediatization trends mentioned earlier in this paper. Live transmissions and online meetings have not only enabled individuals to connect with their community and practice their religion in ways that they had perhaps not previously explored, but also encouraged the Church to engage more in digital media and to be present online more extensively than they used to. This change, since it required making quick adjustments on the side of the Church as well as the followers, brought about various challenges. As one interviewee has said:

This is obviously directed at people who do have the media, yes, such as computers, mobiles phones, or tablets. So, what does it look like at the moment for older people, because maybe not all of them have, although very many actually do have a computer … and we even have a situation where various applications are currently being used so that we can meet together, and, for example, exchange experiences, problems, or pray together. Yes, and even a hundred-year-old person from our church participates in these meetings via Zoom. The pastor set it up for them and they participate, so as far as I know, today probably everyone has either a phone or a computer. (Polish press editor, female)

This quote shows that the switch to digital media has brought to light inequalities related to access to technology and the skills to use it, which meant that some followers were facing involuntary exclusion from the community during the pandemic. The interviewee pointed to elderly Adventists who in some cases do not have access to the internet at home, but the list encompasses also underprivileged Church members who cannot afford a computer or access to a stable Internet connection, or those Adventists with disabilities who cannot make use of digital media without assistance. In some cases, the pastors took on the role of media teachers, instructing the Church members on how to use it, or even installing equipment or software for those who could not do it themselves.

This entrepreneurial attitude and active stance towards exclusions and divides was a new role that many of the Church leaders took on: preventing the segmentation of a religious community required supporting and expanding its technical infrastructure. This entailed that some religious leaders, in the pandemic context, also became *media experts*, who in order to mitigate the digital divides and segmentations, used and asserted their authority by deploying a media-oriented set of skills. This conclusion may evoke Heidi Campbell’s concept of “digital creatives”, i.e. “individuals with specialized skills in computer coding—web or software designers, digital content creators, and/or social media innovators—who leverage their expertise for a specialist cause, often motivated by a personal passion or agenda” (Campbell 2016, p. 7) and gain a special status in their community (cf. Campbell 2021). However, the pastors’ role differs from it substantially, as they acted on behalf of their Church and congregations, and their authority connected their community-orientation, religious agenda, as well as their digital skills. The pastor who taught the elderly Church member to use Zoom may have done so out of personal motivation, however, the ultimate goal of this activity was to ensure that all community members are able to participate. The pastor’s skills were not used to develop the technology itself, but to fulfill institutional goals: technology use was a means to an end.

## Conclusion: What can the COVID-19 pandemic tell us about the connection between media and religion?

The analysis has shown that in response to the COVID-19-related restrictions, the Seventh-Day Adventist Church in Poland deployed several strategies to mitigate the negative impact on the congregations. Apart from communicating with the government and some other minority organizations (like the Evangelical Alliance), it intensified its media productions. The media department employees declared having more awareness of the changing needs of the audiences, and approached the digital media publications strategically, to reach also non-Adventists. The strategy itself is of course nothing new, as the Church media have always supported outreach as well as nurturing, but according to the interviewed specialists, the scale of such activities became larger during the pandemic. In fact, on the YouTube channels of some Adventist congregations, there has been a notable increase in the amount of published content between March 2020 and March 2021, when compared to 2018–2019.

The analysis has also shown that the switch of several Church activities to digital media enabled new forms of participation for the followers. In some cases, it was related to a change in media use habits: the increase in media production was then met with increased media consumption. The extensive use of communication tools, software, and devices within the Church was also conducive to facilitating “doing religion” with the means of digital media. Therefore, in the context of the discriminatory regulations, the intensification of media use was not just last resort, something that had to be endured, but it was a way of keeping the congregation engaged and supported in difficult and uncertain times. At the same time, the switch to digital media had a negative impact on those followers who lacked the skills or the means to make use of it. Therefore, the risk of exclusions and divides among the members of the congregation was much higher in the absence of offline forms of community participation.

To mitigate these risks, some pastors took on the roles of media experts and educators, helping those members of the Church who required assistance in using the digital means of communication. They did so to ensure that the lockdowns and restrictions cause as little disruption to the community as possible. Therefore, under the new circumstances, the pastoral authority out of necessity had to comprise also some degree of technical, digital skills. It should be mentioned that taking on this novel role and responsibility has also been observed in the UK sample, which suggests that the Church as a whole acknowledges the pressure to adjust both to the pace of innovation, and to digital exclusions and divides (by mitigating them). Simultaneously, this new dimension of authority may also be considered a consequence of the Church’s position in the Polish context before the pandemic. The Seventh-Day Adventist Church in Poland, with no access to public television, and very limited presence in the public radio, as well as due to limited resources, had been reliant on its own media infrastructure for decades before 2020. For the Church, using digital media has been one of the ways to participate in the Polish public sphere, which in the religious segment is dominated by the Roman Catholic Church. Perhaps this self-reliance caused the transition to digital media to take place rather smoothly: as many of the interviewees have admitted, the necessity to switch to online communication was not particularly difficult for the Church from the purely technical point of view, because the majority of congregations have already been digitally connected, and their pastors equipped with at least basic digital media skills. In many cases, it is the pastor’s role to manage the social media of the local congregation, post content on its website, etc., which makes having or acquiring some digital competences an important element of ministry and pastoral duties. Furthermore, since the Adventist Church’s international character is often reflected in the fact that pastors serve in different countries throughout their career, digital media use is one of the most efficient means for the pastors to remain connected to their homeland, or family and friends abroad, as well as to access various resources for ministry. In consequence, pastors may also somewhat “naturally” assume the role of digital or technological experts for their congregants, using their experiences and skills to assist those who may need help. It can therefore be concluded that the pandemic became an opportunity for that potential or marginal dimension of authority to come to the fore.

When reflecting on what the lens of pandemic can tell us about the relationship between media and religion, it is worth mentioning that the consequences of deep mediatization may in fact contribute to the transformation of religious organizations, albeit in a more nuanced way than the deep mediatization concept itself may suggest. The intensification of the processes of mediatization which has been observed in the Seventh-Day Adventist Church in Poland indicates a complex interplay between how the consequences of mediatization affect different levels of the Church, and the Church’s responses and mitigatory measures towards the adverse effects. First of all, on the most general level, deep mediatization entails the transformation of communication—publishing videos on social media is a different form of communicating with the followers than taking part in a sermon during church service. The form and content of that communication change, and, as the interviews indicate, the Church media departments are aware of these developments. Positioning videos on the Church YouTube channels is just one example of responding to the changes.

Secondly, the pandemic has shown that deep mediatization may effect in the change of relationship between religious authority and the followers. Because of the need to use digital media, and to react to the trends of deep mediatization, pastors and Church leaders must take on new roles. While their authority is not necessarily undermined by the digital media’s presence in the organization (cf. Cheong 2016; Hoover 2016; Kołodziejska and Neumaier 2017; Kołodziejska, 2018), it may change its form. Rather than being based on assumed positions and statuses (i.e., being a pastor, a minister, etc.), it must incorporate various technical skills that pertain to using various devices, software, and communicating via various media, such as social media, communicators, etc. This development is particularly interesting in view of the concepts of “digital creatives” (Campbell 2016, 2021) and “media pioneers” (Hepp 2016), which point to the digital skills themselves as sources of authority and unequal distribution of power. Both concepts also focus on the individuals, their personal agendas, goals, and know-how: although their qualities may contribute to the development of the organizations (including religious ones), they do not necessarily submit to the goals of these organizations. With the pastors-turned-impromptu-media-experts, it is a different case: while their motivation to help may be personal, the purpose of the activity is to fulfill the organizational goals and to benefit the congregation. Furthermore, putting their skills to use is also connected with religious motivations, in particular those related to maintaining religious community and offering spiritual advice to the followers.

Thirdly, the digital media use among the lay Church members may create a wholly different dimension of community: this is not to say that one form will replace the other, but rather to stress the fact that digital communication may become a legitimate extension of the community-building processes. Although Church leaders may be skeptical at this point, and argue that online gatherings are only an ersatz for the offline ones, the pandemic has shown that at least under some circumstances, this may not be the case. While some elements of “traditional” Church activities cannot be done remotely at this point, perhaps in the future new possibilities will open up. To give one example, the US Presbyterian Church issued an Advisory Opinion in late March 2020, in which online communion was allowed—the argument being that under such extraordinary circumstances, when the health and safety of the followers is at serious risk, Last Supper during streamed online service may be the only way for them to receive the sacrament (Jones 2020). Supported by biblical interpretations, the opinion assumes that virtual communion is a temporary adjustment. However, this precedent may signal that in the future other religious organizations will acknowledge various forms of digital religious practices as theologically justified, legitimate, and equal to offline ones.

## Footnotes

1 The media included the official Facebook page of the Seventh-Day Adventist Church in Poland, the YouTube Channel of Głos Nadziei (Adventist radio and TV producer), and YouTube channels of several local congregations including Warsaw, Gdynia, Łódź, Skoczów, and Poznań. All the channels publish their original content in Polish.

2 The 2021 festival was fully online (via YouTube, Zoom, etc.), and took place in April 2021.

3 https://www.youtube.com/c/Ko%C5%9Bci%C3%B3%C5%82Adwentyst%C3%B3wDniaSi%C3%B3dmegowGdyni/featured [in Polish].

4 https://www.youtube.com/user/ZborSkoczowADS/featured.

5 See press article: https://gs24.pl/abp-andrzej-dziega-sugeruje-ze-w-kosciele-nie-mozna-sie-zarazic-bo-wody-swieconej-boi-sie-diabel-chrystus-nie-roznosi-zarazkow/ar/c1-14858835 [in Polish].
